# Retraction: Computational design and molecular modeling of the interaction of nicotinic acid hydrazide nickel-based complexes with H_2_S gas

**DOI:** 10.1039/d5ra90037a

**Published:** 2025-04-04

**Authors:** Hitler Louis, Daniel Etiese, Tomsmith O. Unimuke, Aniekan E. Owen, Abdulahi O. Rajee, Terkumbur E. Gber, Chioma M. Chima, Ededet A. Eno, Emmanuel N. Nfor

**Affiliations:** a Computational and Bio-Simulation Research Group, University of Calabar Calabar Nigeria louismuzong@gmail.com; b Department of Pure and Applied Chemistry, Faculty of Physical Sciences, University of Calabar Calabar Nigeria; c Department of Chemistry, Akwa-Ibom State University Uyo Nigeria; d Department of Chemistry, University of Ilorin Ilorin Nigeria; e Department of Chemistry, University of Buea Buea Cameroon

## Abstract

Retraction of ‘Computational design and molecular modeling of the interaction of nicotinic acid hydrazide nickel-based complexes with H_2_S gas’ by Hitler Louis *et al.*, *RSC Adv.*, 2022, **12**, 30365–30380, https://doi.org/10.1039/D2RA05456F.

The Royal Society of Chemistry, with the agreement of the named authors, hereby wholly retracts this *RSC Advances* article due to concerns with the reliability of the data.

The NCI plots in Fig. 6 are identical to each other and to images in other papers by the same author group but representing different systems.^1,2^ The authors have not been able to satisfactorily explain this.

In addition, a number of references were inappropriately replaced with self-citations by the authors during the revision process.

Given the significance of these concerns, the Editor has lost confidence that the findings presented in this paper are reliable.

The authors were informed about the retraction of the article Tomsmith O. Unimuke and Abdulahi O. Rajee have agreed with the decision, the other authors have not responded.

Signed: Tomsmith O. Unimuke, Abdulahi O. Rajee

Date: 28th March 2025

Retraction endorsed by Laura Fisher, Executive Editor, *RSC Advances*
